# Advances in Extraction, Structure, and Physiochemical Properties of Sorghum Kafirin for Biomaterial Applications: A Review

**DOI:** 10.3390/jfb15070172

**Published:** 2024-06-24

**Authors:** Umar Shah, Rewati Bhattarai, Hani Al-Salami, Christopher Blanchard, Stuart K. Johnson

**Affiliations:** 1 School of Molecular and Life Sciences, Faculty of Science and Engineering, Curtin University, Perth, WA 6845, Australia; umarshah.umarzahoor@gmail.com (U.S.);; 2 The Biotechnology and Drug Development Research Laboratory, Curtin Medical School and Curtin Health Innovation Research Institute, Curtin University, Perth, WA 6845, Australia; 3 ARC ITTC for Functional Grains, Graham Centre for Agricultural Innovation, Charles Sturt University, Wagga Wagga, NSW 2678, Australia

**Keywords:** kafirin, structure, high hydrophobicity, self-assembly, low digestibility, biomaterial, extraction, purification

## Abstract

Kafirin is an endosperm-specific hydrophobic protein found in sorghum grain and the waste by-product from sorghum biorefineries known as sorghum dried distillers’ grain with solubles (DDGS). Because of kafirin’s poor nutritional profile (negative nitrogen balance, slow digestibility, and lack of some essential amino acids), its direct human use as a food is restricted. Nevertheless, increased focus on biofuel production from sorghum grain has triggered a new wave of research to use sorghum DDGS kafirin as a food-grade protein for biomaterials with diverse applications. These applications result from kafirin’s unique chemical nature: high hydrophobicity, evaporation-induced self-assembling capacity, elongated conformation, water insolubility, and low digestibility. Aqueous alcohol mixtures have been widely used for the extraction of kafirin. The composition, structure, extraction methodologies, and physiochemical properties of kafirin, emphasising its biomaterial functionality, are discussed in detail in this review. The literature survey reveals an in-depth understanding of extraction methodologies and their impact on structure functionality, which could assist in formulating materials of kafirin at a commercial scale. Ongoing research continues to explore the potential of kafirin and optimise its utilisation as a functional biomaterial, highlighting its valuable structural and physicochemical properties. Further studies should focus on covering gaps in the research as some of the current structural understanding comes from data on zein protein from maize.

## 1. Introduction

Understanding protein structure and interactions at the molecular level has a crucial role in biomaterial preparation due to their influence on material’s functionality and performance [1]. Factors like high hydrophobicity, slow digestibility, self-assembling capacity, water insolubility, and surface properties need to be understood in-depth to identify the potential biomaterial applications of specific proteins.

Proteins are classified within many groups with widely different physicochemical properties; however, prolamins, a group of cereal grain storage proteins, are known for their high thermal stability, resistance to enzymatic proteolysis, self-assembling nature, high hydrophobicity, and slow digestibility. Due to these unusual properties, the potential of prolamins such as zein from maize and kafirin from sorghum for biomaterial applications in the food and pharmaceutical industry is currently a topic of exploration [2].

**Figure 1:**
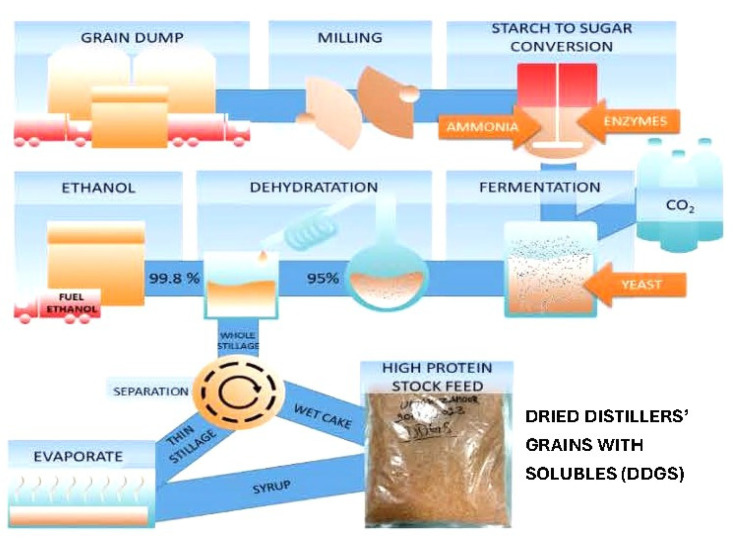
Production process for manufacture of biofuels from sorghum grain resulting in waste product of dried distillers’ grains with solubles (DGGS).

Ongoing research continues to explore the potential of biopolymers from industrial waste such as carbon based nanomaterials from olive solid waste [3] and nanobiochar from orange peel [4] for biomaterial production. The production of biofuels sorghum grain leaves a protein-enriched by-product called dried distillers’ grain with solubles (DDGS) (Figure 1). Until now, there have been no industrial strategies for the use of sorghum DDGS beyond its traditional usage as a low-cost animal feed. New strategies for the use of sorghum DDGS will help to reduce the burden on environmental concerns (as a large amount of sorghum DDGS is dumped in sewers and rivers) as well as add economical value to sorghum grain and the biorefinery process. Sorghum grain and sorghum DDGS contain the prolamin protein kafirin. Kafirin protein contain levels of hydrophobic amino acids, and this hydrophobic-to-hydrophilic amino acid ratio is among the principal characteristics that induce the self-assembly to kafirin into various mesostructures (micro-to-nano metre scale), ranging from spherical particles to fibres. For application as a food- and pharmaceutical-grade biomaterial, kafirin satisfies all key characteristics: generally recognised as safe “GRAS” status [5]; natural origin and biodegradable [6]; slowly digested by proteases; low-cost; and non-allergenic [7].

Various methodologies have been developed and used for the extraction and purification of kafirin protein, including aqueous alcohol mixtures with or without additives (such as reducing agents) [8], glacial acetic acid methodology [9], and purifications via anion and cation exchange chromatography [10]. Despite the advancements in the science of kafirin extraction and purification, there are still some limitations in terms of its potential for commercial scale-up, e.g., low selectivity of extraction solvents and economic viability. This review summarises the structure and composition of kafirin and provides an overview of kafirin extraction and purification methodologies. Recent developments in the physicochemical properties of kafirin proteins with emphasises on material functionality are discussed.

## 2. Protein Composition of Sorghum Grain

Sorghum (*Sorghum bicolor* (L). Moench) is a cereal in the grass family *Poaceae* and ranks as the fifth grain crop worldwide in terms of production after rice, wheat, maize and barley [11]. This high production is related to its resistance to high temperatures, drought, and fungal infections [12]. Sorghum grain is rich in available carbohydrates (mainly starch) (~65% dry basis), protein (~12% dry basis), lipids (~4% dry basis), and dietary fibre (~2% dry basis) and contains valuable levels of minerals, vitamins, and polyphenols [13].

Sorghum proteins are classified into five groups based on solubility criteria: (a) water-soluble albumin; (b) salt-soluble globulins; (c) alcohol-soluble kafirins; (d) alcohol- and reducing-agent-soluble, cross-linked kafirins; and (e) alkaline-soluble glutelin [14]. Among these, kafirin, accounts for ~70% of total protein in the grain [15]. The prolamins are rich in proline and glutamine amino acids (from which the name prolamin is derived) and are found in corn (zein), sorghum (kafirin), oats (avelin), rice (prolamin), rye (secalin), and barley (hordein). Based on their molecular weight and amino acid composition, prolamins are categorised into various subgroups (Table 1).

**Table 1:** Prolamin subunits and their percentage in different grains.

Grain	Prolamin	Prolamin Subunits	Prolamins % of Total Protein	Reference
Sorghum	Kafirin	α-, β-, γ-	70–80	[14]
Maize	Zein	α-, β-, γ-, δ-	40–45	[16]
Oats	Avelin		4–15	[17]
Rice	Prolamin	10-,13-, 16-	3–6	[18]
Rye	Secalin	γ- and ω-	17–19	[19]
Barley	Hordein	β-, k-, δ-, γ-	50–80	[20]

As such, kafirin exists in protein bodies. The protein bodies are specialised organelles within the endosperm cells of sorghum seeds, in which kafirin accumulated during seed development and maturation [21]. These protein bodies containing kafirin typically exhibit a spherical or ellipsoidal shape, ranging from a few micrometres to tens of micrometres in diameter [21]. In these protein bodies, kafirin molecules are densely packed together, forming a matrix like-structure that surrounds and encases lipid droplets. This arrangement provides stability and protection to the kafirin molecules, shielding them from enzymatic activity (proteolytic) and other environmental stresses during seed storage [22]. During the extraction process, the disruption of the native structure of kafirin is necessary to isolate kafirin for many diverse applications including the development of biomaterials. This breakdown typically involves physical, chemical, and mechanical procedures that disrupt the protein bodies of sorghum releasing kafirin molecules from their native environment. Despite their high nutrient content, the use of sorghum grain in the mainstream global food system is restricted because of the low digestibility of its protein and starch and deficiencies in some essential amino acids, such as lysine and threonine [23]. To address these deficiencies, considerable research has been conducted using conventional breeding and genetic modification [14]. Chemical mutagenesis has been used to produce high protein digestibility/high lysine sorghum by suppressing the synthesis of γ-kafirin and β-kafirin, with a concomitant increase in non-kafirin and decrease in kafirin content in the endosperm [24]. Similarly, sorghum protein nutritional quality (in terms of digestibility, amino acid score, and digestibility-corrected amino acid score) was improved by co-suppressing the synthesis of kafirin subunits, i.e., α-, γ-, and δ- kafirin subunits and removal of tannin.

Kafirin was first extracted from a sorghum grain type called “kafir”, from which it gets its name, by Johns and Brewster in 1916 using 70% ethanol, followed by 60% tertiary butyl alcohol [25]. They found that the seeds of kafir contains more than one-half of a protein (kafirin) that resembles the maize protein zein. Its resemblance with zein is based on its elemental composition, for example, kafirin and zein have carbon contents of 55.19 and 55.23, hydrogen contents of 7.36 and 7.26, sulphur contents of 0.60 (both), nitrogen contents of 16.44 and 16.13, and oxygen contents of 20.41 and 20.78, respectively. However, they reported that kafirin contains higher levels of tryptophan and lysine, both of which are lacking in zein.

## 3. Extraction and Purification of Kafirin

Sorghum components gained commercial attention in the USA during World War II due to the shortage of maize. From 1948 to 1970, Corn Product Co. (Corpus Christi, TX, USA) used sorghum to produce starch and dextrose. However, its use was discontinued due to (a) low starch recovery; (b) low oil yield; (c) high wax content; (d) the price of sorghum rising to almost that of maize [26].

An early method used for laboratory-scale sorghum protein isolation was sequential (Osborne) extraction [27], in which water-soluble proteins (albumins) and salt-soluble proteins (globulins) are extracted by water and salt solutions, respectively, followed by extraction of prolamins by aqueous alcohol at room temperature. Other early methodologies for extracting kafirin include the Landry–Moureaux method, which extracted kafirin fractions with different molecular weights, either with alcohol or aqueous alcohol and reducing agents like sodium metabisulphite. Using this approach, the extracted protein fractions were as follow: Fraction I, albumins and globulins extracted in the presence of NaCl; Fraction II: Type-1 kafirin extracted using 60% butanol (aq); Fraction III: type-2 kafirin using 60% butanol (aq) plus the reducing agent, mercaptoethanol; Fraction IV: glutelin-like protein using alkali borate buffer and mercaptoethanol; and Type V: true glutelins extracted with alkali borate and sodium dodecyl sulfate.

Glacial acetic acid [9] and chromatographic purification [10] have been reported for extraction and purification of kafirin (Table 2). In comparison to zein, kafirin requires a lower-polarity solvent because of its more highly hydrophobic nature. Tertiary propanol [28], sonication + sodium borate [29], reducing agent + high temperature + sonication, and 2-propanol [30], reducing agent [31], high temperature [8], and reducing agent + high temperature [31] are all approaches that have been successfully used for kafirin extraction. Reducing agents are particularly desirable to extract the polymeric components of kafirin, but monomeric and oligomeric kafirin subunits may not need reducing agents for extraction. Further, different subunit types have different solubility when compared at the same polymerisation level and in the same solvent system. β-kafirin, for instance, demonstrates lower solubility in aqueous alcohol than the other types due to its higher level of disulphide bonding between monomers.

Kafirin protein is highly non-polar but contains some polar amino acids; thus, extraction solvent systems should possess mixed characteristics, i.e., the capacity to solvate ionic polar, non-ionic polar, and non-polar groups. A primary solvent alone dissolves kafirin protein at solvent concentrations of >10%. Examples of primary solvents include aqueous acetamide, 2-amino-2-ethyl-1,3-propanediol, butylamine, glacial acetic acid, phenol, and ethylene glycol in water [32]. Each primary solvent needs a different temperature and solvent concentration for maximum kafirin dissolution. For example, propylene glycol (aq) is effective at room temperature, but ethanol (aq) and glycerol (aq) need elevated temperatures of 60 °C and 150 °C, respectively.

Binary solvents are those in which a second component is added to either water or a lower-molecular-weight aliphatic alcohol, such as ethanol and methanol—for example, (a) water + acetone, n-butanol, ethanol, isopropanol, or methanol; (b) a lower aliphatic alcohol + ethyl lactate, ethylene glycol, furfural, methylene chloride, or propylene glycol. In these binary systems, the more polar solvent, e.g., water, interacts with polar amino acids. In contrast, the less polar organic compound interacts with the non-polar amino acids to give effective dissolution [27].

**Table 2:** Methodologies for kafirin extraction and the resulting protein content, yield, and extraction details.

Authors (Year)	Yield (%)	Purity (%)	Miscellaneous Details/Pre-Treatment	Extraction	Process Flow
[33]	19	16.6	Albumin, globulin and LMW N2 fragments	(1) An amount of 0.5 M NaCl for 60 min.	Sequential extraction in given order.
	0.3	NA	Salt and traces removal	(2) Double distilled water for 20 min.	
	11.6	17.3	Kafirin	(3) A total of 60% 2-propanol for 4 h.	
	20.8	24.5	Cross-linked kafirin	(4) A total of 60% 2-propanol + 1% dithioreitol (DTT) for 4 h.	
	3.8	4.8	Glutelin like proteins	(6) A total of 0.1 M borate buffer, pH 10.8 for 4 h.	
	44.5	27.2	Glutelin	(5) A total of 0.1 M borate buffer, pH 10.8 + 1% DTT + 1% sodium dodecyl sulphate (SDS) for 18 h.	
		11.5	Non-extractable nitrogen		
[34]	46.1 (WGF)	NA	Adaptation of Carter and Reck process [35].	A total of 70% *w*/*w* ethanol in distilled water, 0.35% *w*/*w* sodium hydroxide, and 0.5 *w*/*w* sodium metabisulphite at a ratio of 1:5 *w*/*w* flour-bran to extraction with vigorous stirring at 70 °C for 1 h.	Extraction, centrifugation, evaporation, pH, precipitation, freeze-drying, and oil removal.
[9]	54.3	89.3	Comparison of different extractants; flour screened through 800 µm screen.	A total of 70% ethanol + 0.5% sodium metabisulphite + 0.2% NaOH at 70 °C.	Extraction, centrifugation, evaporation, pH, precipitation, filtration, freeze-drying, and oil removal.
	55.3	91.2		A total of 55% isopropanol + 0.5 sodium metabisulphite + 0.3% NaOH at 40 °C.	
	25			Glacial acetic acid at 25 °C.	
	25			Glacial acetic acid + 0.5% sodium metabisulphite at 25 °C.	
	52.8			Presoak (1 h) 0.5% sodium metabisulphite; glacial acetic acid at 25 °C.	
	59.3			Presoak (16 h) 0.5% sodium metabisulphite; glacial acetic acid at 25 °C.	
	61	92.9		Presoak (16 h) 1.0% sodium metabisulphite; glacial acetic acid at 25 °C.	
[8]			DDGS washed with hot water (50 °C); dried in hot-air oven at 50 °C overnight before extraction	A total of 70% (*w*/*v*) ethanol + 0.5% *w*/*w* sodium metabisulphite + 0.35% *w*/*w* NaOH at 70 °C for 1 h.	Hot water wash, oil removal, extraction, centrifugation, dilution, centrifugation, deionised wash, and freeze-drying.
[36]	87 (total protein)		Flour defatted with n-hexane (1:10 ratio); washed with water (1:10 ratio) for 1 h and centrifuged at 8000 rpm for 10 min. The procedure was repeated with 0.5 M NaCl and distilled water.	Extracted with 60% t-butanol for 2 h each and 10 min of ultrasonication using an FS-28 solid-state ultrasonicate (bath-type with sonic power, 225 W; sweep frequency, 40 kHz) at interval of 30 min.	Oil removal, saline wash, extraction, centrifugation, evaporation, pH precipitation, filtration, and freeze-drying.
[37]	70 (total protein		Adapted from [34].	Sorghum flour (250 g) extracted using a mixture of 900 mL ethanol (70% *w*/*w*) in deionised water, 25 g/kg sodium metabisulphite, and 17.5 g/kg *w*/*w* sodium hydroxide as a reducing agent. The mixture was heated and held at 70 °C with continuous stirring for 1 h.	Extraction, centrifugation, dilution, freeze-drying, milling, and oil removal.
[38]	54.3	67.2	Percolation (liquid-to-solid ratio of 2:5:1).	A total of 70% *w*/*w* aqueous ethanol + 1.0% *w*/*w* sodium metabisulphite + 0.35% *w*/*w* NaOH at 70 °C for 1 h.	Extraction, evaporation, pH precipitation, dilution, filtration, and air-drying.

Tertiary solvent systems are those with a combination of water, low aliphatic alcohol, and other components. Examples reported for kafirin extraction include water + lower aliphatic alcohol + acetone, ethylene glycol, formaldehyde, or nitromethane [27]. The different extraction systems used for kafirin will now be reviewed in more detail.

### 3.1. Extraction by Glacial Acetic Acid

This food-compatible methodology utilises glacial acetic acid (primary solvent) to solubilise kafirin protein from the sorghum matrix. Glacial acetic acid disrupts protein–protein and protein–non-protein interactions within the sorghum matrix, releasing kafirin into the acetic acid. The acidic conditions created by the glacial acetic acid help to solubilise kafirin by protonating ionisable amino acid residues, thereby enhancing the solubility of kafirin in the acetic acid [9]. This protonation of ionisable amino acid residues reduces the net charge by weakening strong electrostatic interactions, increasing hydrogen bonding and ion-diploe interactions; subsequently, this enhances the solubility of kafirin in polar glacial acetic acid solvent.

The glacial acetic acid was used as a primary solvent with and without reducing agent to extract kafirin from sorghum flour [9]. The authors found it desirable to presoak sorghum flour in 0.5% sodium metabisulfite for 16 h before extraction to provide a better yield. The extracted kafirin showed the presence of all major kafirin subunits. The glacial acetic acid without a reducing agent gave a lower yield than that of glacial acetic acid with sodium metabisulfite overnight soaking. The use of sodium metabisulfite may have reduced the high levels of disulfide cross-linking between the kafirin subunits, as well as disrupting kafirin starch interactions. So, this technology is primarily based on the selective solubilisation of kafirin in an acidic environment, which is facilitated by the denaturation and solubilisation effects of glacial acetic acid. This methodology offers various advantages, including food compatibility, cost-effectiveness, high efficiency, versatility, and compatibility with downstream applications [14].

The structure and physicochemical properties of kafirin protein made from sorghum DDGS and extracted using three methodologies including glacial acetic acid methodologies were investigated [39]. The results from the study revealed that the protein content and yield of kafirin were higher when acetic acid (44.1% yield and 98.9% protein content) and alkaline-ethanol methodologies (56.8% yield and 94.9% protein content) were employed compared to the acid ethanol methodology (24.2% yield and 42.32% protein content). FTIR analysis of the extracted kafirins determined that the acetic acid and the alkaline-ethanol kafirins contain greater levels of α-helices and random coils than that of kafirin made from the acidic-ethanol methodology, which contained more β-sheet conformation. This presence of the β-sheet in kafirin isolated using the acidic-ethanol methodology could be due to the higher extraction temperature employed in this treatment. Size exclusion chromatography revealed that the acetic acid and the acidic-ethanol extraction methodology extracted higher-molecular-weight polymeric proteins than the alkaline-ethanol methodology. Notably, the results from chromatography revealed the presence of γ-kafirins only in the alkaline-ethanol methodology. The use of glacial acetic acid is considered superior because it is a food-compatible solvent, non-volatile, and does not require a licence or raise regional concerns, as is the case with alcohols. The only restriction to this extraction solvent is that the solvent temperature should be kept low to prevent polymerisation of kafirin polypeptides. Careful selection of extraction conditions, such as the concentration of glacial acetic acid, time-temperature combination, and pre-soaking conditions (with and without reducing agent), could achieve better yields of kafirin with minimal interference from other sorghum components.

### 3.2. Extraction by Aqueous Alcohol Methodology

Kafirin is insoluble in water alone due to its high hydrophobic nature. Therefore, aqueous alcohol mixtures (secondary solvent) can effectively and selectively (specific fractions of kafirin) extract kafirin from the sorghum matrix. The principle behind the extraction of kafirin using water-alcohol mixtures lies in differential solubility [32]. In a water–alcohol mixture, alcohol serves as a protein denaturant and solvent, disrupting protein–protein interactions and interactions with other sorghum components such as starch. Water helps to maintain the solubility of polar components including polar amino acid residues such as cysteine, tyrosine, glutamine, etc., for protein extraction [32,40]. Several laboratory-scale studies have extracted kafirin using aqueous alcohol mixtures, with high extraction yields for use in various downstream applications such as structural, physicochemical, and biochemical analysis, as well as food science research [8,39]. Compared to acetic acid, this methodology offers higher purity advantages [41], primarily because alcohol can disrupt the non-covalent interactions like hydrogen bonding, ionic factors, and van der Waals interactions of the kafirin more effectively.

The sorghum prolamin kafirin and sorghum non-prolamins have been differentiated using the extract methodology that was previously applied to maize [42]. Sorghum flour was first treated with sodium chloride (aq) to remove albumins and globulins. The resulting insoluble residue was then treated with an aqueous solution of sodium borate, sodium dodecyl sulfate, and mercaptoethanol at pH 10 with a sorghum flour to solvent ratio of 1:10. After 1 h, the samples were centrifuged. Then, 60% butanol was added to the supernatant to precipitate the non-kafirins. Then, the supernatant that contained kafirin was separated from the pellet. A similar procedure was followed by [43] to identify the parameters and factors required for sorghum protein extraction to minimise extraction time. This optimised extraction study revealed that the sorghum flour to solvent ratio, pH, detergent and reducing agent were significant factors affecting sorghum protein extractability. For example, at pH 2.5 to 10, the extraction yield increases with increased pH in the presence of sodium dodecyl sulfate.

An ultrasound-assisted tert-butanol methodology was used to isolate and purify kafirin [41]. These authors milled sorghum to 40 mesh to break open the cellular structure and to achieve uniform particle size, followed by defatting using n-hexane. The defatted sorghum flour was extracted with water and 0.5 M NaCl, followed by centrifugation. In this way, water-soluble protein (albumins) and salt-soluble protein (globulins) were removed by discarding the supernatant. The remnant was then used for isolation and purification of kafirin using tert-butanol (60%) twice for 2 h, followed by ultrasonication (10 min) and centrifugation; the supernatant was freeze-dried directly. The authors reported a protein content of 87% dry basis and extraction of all major kafirin polypeptides.

Most of these methodologies for kafirin extraction and purification have some problems in terms of commercial scalability including cost, toxicity, and safety concerns. For example, solvents such as tert-butanol are highly toxic, ethanol is flammable, and its use is prohibited in some regions of the world. However, water–alcohol plus reducing agent remains popular for the extraction of kafirin at the laboratory level due to high yield, high assembling capacity, and easy solvent recovery.

### 3.3. Ultrasonic Assisted Extraction

Ultrasound-assisted extraction utilises high-frequency sound waves to disrupt the cell structure of sorghum grain, leading to the release of kafirin. This process involves the generation of cavitation bubbles in the solvent, which implode due to the alternating cycles of compression and rarefaction produced by the sonic waves. This phenomenon creates localised regions of high pressure and temperature that lead to the physical disruption of cell walls and membranes. As a result, kafirin protein is released from the endosperm matrix and becomes solubilised in the extraction medium. Ultrasound-assisted extraction offers some advantages for kafirin production, including higher efficiency, reduced extraction time, and enhanced mass transfer compared to the water–alcohol only methodology [44].

Ultrasound-assisted extraction of sorghum flour was followed by size exclusion and reverse-phase, high-performance liquid chromatography (HPLC) analysis [45]. A sorghum meal slurry containing sodium borate (pH 10) and sodium dodecyl sulfate was sonicated at 10 watts for 20 s. Sonication was seen to reduce the molecular weight of large proteins by breaking non-covalent bonds through shear degradation. Sonication was seen to extract more sorghum polymeric protein than sodium borate alone. Sonication might have resulted in enhanced mass transfer, improved solvent penetration, and accelerated diffusion, resulting in extraction of polymeric protein. In another study, ultrasound-assisted extraction and purification of kafirin were performed [46]. This methodology used heptane for defatting of the sorghum flour; then, albumins were extracted from the defatted sample using water at ambient temperature and the residue was recovered via centrifugation. Globulins were extracted from the residue 0.5 M NaCl (aq) followed by centrifugation of the kafirin-containing residue, which was then lyophilised. The lyophilised residue was sonicated in water at different amplitudes, 0%, 20%, and 40%, for different time combinations (5 min or 10 min) at 4 °C at a frequency of 20 kHz. The sonication treatment to the samples was followed by centrifugation, and kafirin was isolated from the residue using 60% isopropanol at room temperature. The kafirin pellets were recovered via evaporation of the solvent. Sonication treatment gave kafirin of a higher yield compared to non-ultrasonicated control samples. Furthermore, sonication altered the secondary structure of kafirin, as evident from CD and FTIR spectroscopy. Some limitations associated with ultrasound-assisted extraction include difficulty in upscaling and higher equipment costs.

### 3.4. Chromatographic Purification of Kafirin Protein

Chromatographic purification of proteins is one of the most efficient technologies in biochemistry for purifying proteins from complex mixtures. It exploits differences in the physicochemical properties of proteins to separate them based on their interactions with a stationary phase (solid or liquid) and a mobile phase (liquid). This technology offers advantages such as high purity, selective separation, automation, and compatibility with downstream applications.

Adsorptive chromatography was used for the purification of kafirin, providing the first scientific insight into the isotherm and kinetic studies of kafirin adsorption on anion- and cation-exchange resins for future practical application in preparative scale chromatography [10]. In this methodology, kafirin extraction was performed via steeping and fractionation of sorghum seeds to obtain endosperm and bran fractions. In brief, 100 g of sorghum was soaked in 200 mL distilled water containing 0.3% *w/v* sodium metabisulphite and 1.4% *v/v* lactic acid and steeped for 48 h at 50 °C under stirring conditions (200 rpm). Further extraction steps in this processing followed those from the literature [34], using 70% absolute ethanol in distilled water containing 0.1% sodium metabisulphite and 0.1% sodium hydroxide at 50 °C for 1 h. The author’s reported that the extracted protein had impurities such as triglyceride oil, free fatty acids, and traces of phenolics. Such impurities influence the higher material functionality of the protein kafirin such as in the preparation of prolonged release delivery systems. Generally, phenolic impurities might be easily removed by washing with the 30–35% ethanol, but the oil remains with kafirin, possibly due to its hydrophobic association with kafirin, when it is precipitated at its isoelectric point. Likewise, hexane wash removes oils but with associated limitations such as toxicity and a flammable nature. The study indicated that, given the solubility of kafirin in aqueous ethanol, a preparative adsorption system is of interest. The authors found that the differences in surface chemistry, hydrodynamic design of the adsorbent, and the surface charge of kafirin with respect to the environment in batch equilibrium experiments as well as the uptake kinetic experiments were significant contributors in determining the adsorption behaviour. Kafirin adsorbed on anion exchange resins can be eluted at a pH close to pI. This study is essential to develop and upscale the adsorptive chromatographic processes for the purification of kafirin. Furthermore, the results of SDS-PAGE showed kafirin polypeptides in the range of 13 to 37 kDa consisting of α-kafirin (26–27 kDa), β-kafirin (20 kDa), and γ-kafirin (13 kDa).

## 4. Kafirin Structure

### 4.1. Primary Structure

Kafirin proteins are rich in the hydrophobic amino acids glutamine, proline, alanine, and leucine and are deficient, from a nutritional perspective, in lysine and threonine (Table 3) [41].

Non-polar amino acids comprise 60.1% of total amino acids [41]. Based on molecular sequencing and electrophoretic studies, kafirin has been divided into four groups: α-kafirin subunits, β-kafirin subunits, γ-kafirin subunits and δ-kafirin subunits (Table 4) [10].

**Table 4:** Kafirin protein subunits, molecule weight identification on electrophoretic studies, amino acid composition, and percentage of subunits in total kafirin.

Kafirin Subunit	Molecular Weight (kDa), Amino Acid Residues	Amino Acid Composition	Percentage in Total Kafirin	Reference
*α*-kafirin (*α*1- and *α*2 kafirin)	22–25; 240–250	High in non-polar amino acids and 1 Tyrosine; lysine deficient	80–84	[6,50]
*β*-kafirin	18; 172	High in methionine, cysteine, and 2 tyrosine	7–8	[51,52]
*γ*-kafirin	27; 193	High in proline and cysteine; no lysine and aspartic acid	9–12	[53]
*δ*-kafirin	-; 114	High in methionine; one tyrosine	-	[14,50]

The major component of kafirin, α-kafirin (analogy to α-zein), is high in non-polar amino acids, containing one tyrosine, no lysine, and ten blocks of repeated amino acids that are rich in glutamine, proline, alanine, and leucine. From the electrophoretic profile under the reducing conditions of α-kafirin, Belton et al. [50] first reported that α-kafirin accounted for about 45% of total kafirin, with α1-kafirin at 19 kDa contributing ~25% of total protein and α2-kafirin at 22 kDa contributing ~20% of total protein. Later, it was reported that polypeptides of ~23.8 kDa and ~25.1 kDa corresponded to α1- and α2-kafirin and it was calculated that α-kafirin was 68% of the total kafirin (Table 4) [41]. This shows there is some variation in the data on kafirin composition, which may be related to the variety (genotype) of the sorghum and its production environment. Alpha-kafirin is water-insoluble and tends to form gel-like structure upon hydration, due to its strong hydrophobic interactions. It primarily contributes to the formation of core structure of kafirin protein [54]. The amino acid composition of the α-kafirin at 22 kDa is tabulated in Table 5.

The β-kafirin, having a molecular weight of 17 kDa, accounts for about 7–8% of total kafirin from sorghum varieties with a vitreous (glassy) endosperm and 10–13% in those varieties with an opaque (floury) endosperm [22]. Β-kafirin is high in methionine and cysteine and contains two tyrosines (Table 4). The amino acid sequencing of β-kafirin shows some analogy with β-zein; however, it has ten cysteine residues, while the latter has only seven [50]. β-kafirin tends to behave similarly to α-kafirin in terms of solubility properties—insoluble in water and forming a gel-like structure upon hydration.

The only subgroup readily soluble in water is γ-kafirin; therefore, it is also called “reduced soluble kafirin” [36]. It contains a significant amount of proline, cysteine, aspartic acid, and no lysine. γ-kafirins are found in lower quantities and play a role in the modulation of protein body properties, e.g., porosity and texture. Both β- and γ-kafirins contain higher levels of cysteine than α-kafirin subunits [54].

The δ-kafirin subunits are resolved at ~14 kDa and 21 kDa. The δ-kafirins are rich in methionine, with a methionine content of 26.9 mol % at ~14 kDa and 22.8 mol % at 21 kDa. They are present in trace amounts within the protein bodies and are believed to have regulatory functions.

### 4.2. Secondary Structure of Kafirin Protein

The secondary structure of kafirin has been investigated using the powerful analytical technique of Fourier transform infrared spectroscopy (FTIR) from which it has been elucidated that kafirin polypeptides, in general, are in their native state, highly folded with a secondary structure dominated by α-helices at 40–60% of the total kafirin secondary structure, β-sheet secondary structure at ~27%, and unordered/disordered structures (mixture of random coils) at ~24% [36]. There are only limited secondary structure studies on the individual kafirin subunits. α-kafirin subunits have been reported to contain a greater number of α-helices than other kafirin subunits, as identified using FTIR, while γ-kafirin has more random coils and β-sheet secondary structure than α-kafirin subunits [36]. The α-helical structure in kafirin contributes most highly to its hydrophobicity since the hydrophilic amino acid side chains are closely packed and buried inside the helices, whereas the hydrophobic side of aliphatic amino acids point outwards [56]. In contrast, the β-sheet secondary structure is a more unfolded open structure with hydrophilic amino acid residues present more on the outside of the structure [57].

The proportion of the different secondary structures of isolated kafirin is influence by the solvent systems used for its extraction. For example, dissolution in 60% tert-butanol (aq) gave 58% α-helix, whilst 65% isopropanol (aq) gave 53% α-helix and 85% ethanol (aq) showed 68% α-helix [41]. In-depth understanding of the conformational changes with processing conditions and solvent system, therefore, can help to assist in the development of newer methodologies, to optimise functional properties related to the secondary structure of kafirin. More studies are needed in the near future to investigate the secondary structure of each subunit of the kafirin.

### 4.3. Tertiary Structure of Kafirin Protein

The current understanding of kafirin tertiary structure behaviour is primarily interpolated from data on zein, so direct data from kafirin is clearly lacking in the literature. A summary of zein tertiary structure is, therefore, given here. Based on the circular dichroism study, the model of the α-zein tertiary structure has been proposed as “a group of nine antiparallel α-helices arranged in a cylinder wherein hydrophilic amino acids are on the surface and hydrophobic amino acids are hidden inside” [57]. They proposed that α-helices were highly clustered to form a distorted cylinder in which hydrogen bonds facilitate packing. Based on the small angle X-ray scattering (SAXS) modelling results, combined with some assumptions from the previous literature, we proposed an elongated prism-shaped tertiary structure model, 13 (L) × 1.2 (W) × 3 (D) nm, for zein with 9–10 helical segments aligned in an antiparallel fashion [58]. The assumptions made from the previous literature and added to this proposed model include the follow: each tandem repeat unit (sequence of amino acids) forms a single α-helix and they are joined by glutamine-rich turns or loops; in solution, the secondary structure of zein has 50% α-helix and tandem repeat units favour α-helix [57,59,60].

In terms of tertiary structure, both zein and kafirin are rich in repetitive sequences of amino acids, which contribute to their unique properties. The tertiary structure of kafirin is not as extensively studied as zein, but it is generally believed to consist of a compact structure stabilised by disulfide bonding [36]. The only model proposed for the tertiary structure of purified kafirin is an elongated conformation with dimensions of 11.8 (L) × 1.5 (W) × 1.5 (D) nm and 10 (L) × 1.1 (W) × 1.1 (D) nm in 60% tert-butanol (aq) and 65% isopropanol (aq), respectively [41]. Studies have reported that, on increasing the concentration of kafirin in the solvent, a concentration-dependent aggregation of kafirin occurs as indicated by an overall increase in the radius of gyration with a decrease in the cross-section radius of gyration measured using small angle X-ray scattering (SAXS).

Kafirin from DDGS, which has been exposed to heat treatment during fermentation and drying, has a larger radius of gyration compared to that of kafirin from the raw grain [31]. This indicates the difference in the tertiary structure between the two kafirins, which might be a result of partial unfolding brought about by the exposure to high heat in the case of DDGS kafirin.

Although, the precise quaternary structure of kafirin has not been extensively studies, the formation of disulfide bonds between cysteine residues present in the protein sequence of kafirin is among the most studied cross-linking in kafirin. When two cysteine residues from different kafirin subunits come into close proximity they undergo oxidation to form a disulfide bond, covalently linking the two subunits together [14,36,50]. This process contributes to the stabilisation of overall structure of kafirin including its quaternary structure. Furthermore, the kafirin molecules can assemble through various interactions including hydrophobic interactions and other non-covalent interactions such as capillary and hydrogen bonding and van der Waals interactions.

## 5. Physicochemical Properties of Isolated Kafirin Related to Its Potential Biomaterial Applications

The physicochemical properties of kafirin protein underscore its remarkable versatility and potential in the field of biomaterials. Kafirin is emerging as a potential candidate for biomaterial production due to its inherent biocompatibility, slow digestibility, high hydrophobicity, and surface-active properties [8,14,31,36]. Furthermore, the kafirins assemble into the spherical particles via the evaporation-induced self-assembling mechanism [36], which further serves as a driving force to extensively study the properties of the kafirin for potential biomaterial applications. The following sections will present some of the efficient physicochemical properties of kafirin protein related to their biomaterial applications.

### 5.1. Hydrophobicity

The word “hydrophobicity” is derived from the Greek word “hydro”, which means water, and “phobicity”, which means a lack of affinity. Hydrophobicity plays a key role in kafirin behaviour. Interest in the hydrophobicity of kafirin has been focused on its contribution to the stabilisation of molecular structure. Hydrophobicity can be defined as the excess of free energy of a solute dissolved in water compared to that in an organic solvent under similar conditions. Hydrophobicity is measured based on the free energy of hydration (Δ*G*_sl_) [61].

The hydrophobicity of kafirin is attributed to the presence of hydrophobic amino acid residues and lower negative free energy of hydration. Low negative free energy of hydration implies that the interaction between kafirin molecules and water molecules on hydration releases more energy. To achieve minimum free energy in the folding of protein, non-polar (hydrophobic) groups should be restricted to the interior of folded molecules [62]. The less negative the hydration energy, the greater a protein’s hydrophobicity, and vice versa. The hydration energy of α-kafirin is −144 kcal/mol, β-kafirin is −123 kcal/mol, and γ-kafirin is −100 kcal/mol [23]. Based on the calculated free energy of hydration, γ-kafirin has the highest hydrophobicity [50] despite being the only kafirin subunit soluble in water. The current understanding is that γ-kafirin has significantly higher levels of histidine than other kafirin subtypes. The side chain of the histidine has a pKa of six at the pH of water, providing a higher degree of ionisation, which might cause electrostatic repulsions and, therefore, solubility in water.

The α-kafirin and α-zein have the same level of hydrophobicity, while γ-kafirin has higher hydrophobicity than γ-zein [23]. It is noteworthy that, with the help of an external agent (e.g., heating), kafirin can hydrate more compared to non-heat-treated kafirin. Heating does not change the amino acid content of kafirin, but some conformational changes like the conversion of α-helices into β-sheets due to the effect of heating might result in more water absorption [63].

The surface hydrophobicity of the prolamin proteins is important for biomaterial applications. Broadly, biomaterials are classified into hydrophobic (water-repelling) and hydrophilic (water-loving) and these measurements have previously been performed using the water contact angle technique. The principle of these measurements is that a surface is hydrophobic when its static water contact angle θ > 90° and hydrophilic when θ < 90°.

The surface of zein films exhibited a water contact angle of 126°, showing a predominantly hydrophobic surface [64]. Energy dispersive spectroscopy (EDS) showed that the surface of zein films with 1-octanethiol had a dominance of carbon (78%), oxygen (14.44%), and sulphur (2.27%) elements, indicating that the surface is made of zein elements. They further validated the results of EDS using water contact angle measurements, which showed θ of 115° for zein film while 1-octanethiol had an angle θ of 100°, indicating a high-hydrophobic surface. The authors proposed hydrophobic zein film can be prepared using solvent-induced self-assembly. Understanding the hydrophobicity of kafirin is among the fundamental properties that influences its structure, solubility, functionality, and application in biomaterial fabrication.

### 5.2. Self-Assembly

Kafirin self-assembles in aqueous systems depending on the solvent type, solvent concentration, ionic strength of solution, and temperature. Self-assembly is a process by which a disordered system is converted into an organised structure without any external stimulus, but it relies on weak interactions such as van der Waals and capillary and hydrogen bonds. Both kafirin and zein exhibit an evaporation-induced self-assembling mechanism [36,65]. The evaporation-induced self-assembling mechanism is a process that involves two or more solvents, and one of these solvents evaporates faster. As a result, the polarity of the solution system increases, and this change drives the self-assembly of the protein [65]. This means that in evaporation-induced self-assembly the formulations of kafirin in aqueous alcohol systems undergo an increase in the polarity of the solvent, as alcohol tends to evaporate faster than water. This increased polarity might be the principal driving force for kafirin particle formation. The current proposed mechanisms of kafirin self-assembly behaviour mostly rely on knowledge of zein; therefore, the summary of zein self-assembly mechanisms is given here.

**Figure 2:**
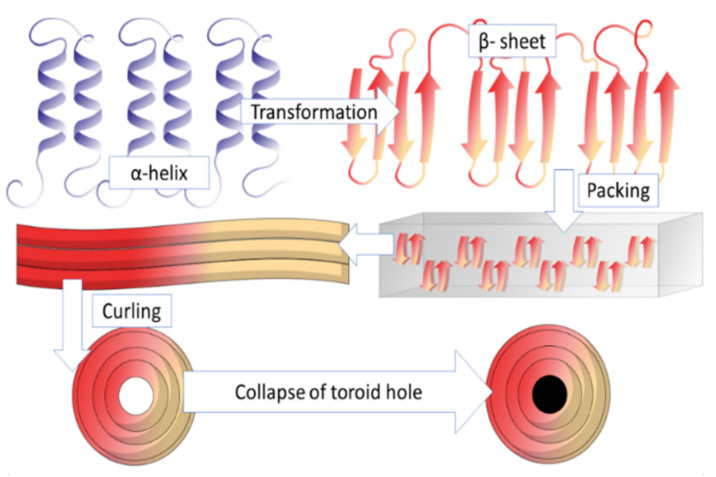
Wang and Padua mechanism for self-assembly of zein. Adapted from Ref. [51].

A novel interpretation of zein particle formation into spheres was performed [66]. They interpreted zein images taken using high-resolution transmission electron microscopy (TEM) and suggested that β-sheets were assembled into strips, rings, discs, and spheres. It was observed during evaporation that the solvent became highly hydrophilic and solvent-induced conformational transitions from α-helices into β-sheet strands may have occurred. After conformational transitions, antiparallel β-sheets packed side-by-side, forming a long ribbon-like structure. The authors proposed that hydrophobic interactions drove β-sheet packing. In the third step, a long ribbon-like structure curled into the ring (toroid) in the centre of the ring was less dense than the edges, suggesting β-sheet lattices at the centre lost regular packing orientation (Figure 2). The ring was seen to enlarge with the addition of new β-sheet strands. Various other studies have been proposed for self-assembling mechanisms of prolamin protein at the micro/nano scale, predominately based on evaporation-induced self-assembly formation [41,65,67].

The formation of zein particles has been proposed as “zein being amphiphilic in nature and have a higher tendency to self-assembly in aqueous phases based on a balance between inner hydrophobic to outer hydrophilic segment” [68]. This formation of zein particles is based on the order of confirmation of minimal energy. Although, like zein protein, there is no clear understanding of kafirin protein in terms of self-assembly. However, considering circular dichroism (CD) spectroscopy analysis [41], it is evident that the kafirin protein exhibits solvent-induced conformational transformation, e.g., a decrease in α-helices on the increasing polarity of the solvent.

### 5.3. Digestibility

The low digestibility of the kafirin plays a pivotal role in determining its suitability for biomaterial fabrication. The possible causes of the low digestibility of kafirin are broadly classified as endogenous and exogenous factors. The endogenous factors (which regulate kafirin functionality) include high disulphide cross-linking [33] and racemisation and isopeptide formation [69]. The exogenous factors include interaction with a range of non-protein ingredients like polyphenols (e.g., tannins), phytic acid cell wall components, and starch [70]. These factors vary considerably depending on the kafirin extraction method and sorghum form used for extraction.

Kafirin from whole grain vs. decorticated grain: Kafirin extracted from the decorticated grain exhibits higher digestibility compared to those extracted from whole grain. This is because the decorticated sorghum has had the outer layer removed, which might decrease the presence of certain anti-nutritional factors or indigestible components that could interfere with extracted kafirin [23].

Kafirin treated with reducing agent: An improvement in sorghum protein digestibility has been reported upon treatment with 2-mercaptoethanol [71]. The cooking kafirin protein in presence of 2-mercaptoethanol increased protein digestibility in vitro with pepsin and trypsin/chymotrypsin. The mercaptoethanol might have led to redcution of disulfide bonds, thereby allowing unfolding the kafirin and making it more susceptible to enzymatic digestion.

Kafirin extracted from grain with high tannin content vs. tannin free: some sorghum genotypes contain high levels of polyphenols, including tannins, compared to other cereals. Co-extraction of kafirin with tannins gives kafirin with reduced digestibility due to the formation of complexes (tannin-kafirin complexes), making kafirin less accessible to digestive enzymes and leading to reduced digestibility. Furthermore, tannins inhibit digestive enzymes such as trypsin and pepsin. This enzyme-inhibition activity of tannin could lead to reduced breakdown of kafirin into smaller peptides and amino acid, leading to reduced digestibility.

Kafirin from transgenic varieties: Studies have reported alterations in kafirin synthesis by suppressing the γ-kafirin using the genetic engineering technique [72]. The authors reported that total kafirin extracted using 60% tert-butyl alcohol from this transgenic sorghum has higher digestibility, probably because of lower levels of disulfide bonding (i.e., less polymerisation via disulfide bonding). Also, the co-suppression of synthesis of major kafirin subunits to improve the overall digestibility of sorghum protein has been investigated [73]. They suppressed two genes (γ-kafirin-1 and γ-kafirin at 25 kDa and 50 kDa), resulting in an increased digestibility profile in vitro. Upon further suppression of α-kafirin (A1 at 25 kDa), they reported a significant increase in protein digestibility. However, low digestibility of kafirin protein enables the development of biomaterial with enhanced stability, prolonged release kinetics, and improved bioavailability.

### 5.4. Solubility

Solubility of kafirin at different levels of structures involves breaking down hierarchical levels of organisation at the molecular and status structures. The ratio of hydrophobic to hydrophilic amino acid residues, folding patterns, and molecular geometry, including molecular weight, influence the solubility of kafirin. Kafirin tends to form aggregates in water due to high hydrophobic attractive forces than repulsive forces. The insolubility of kafirin in water is due to the high proportion of non-polar amino acid residues. The large number of hydrophobic amino acids (i.e., proline, leucine, isoleucine, and phenylalanine) in kafirin and their interactions favour solubility in binary solvents such as ethanol–water and methanol–water [26]. The use of reducing agents can increase kafirin solubility. The reducing agent weakens the kafirin network through cleavage of the di-sulphide bonds with an overall decrease in average molecular weight of protein aggregates [74]. It is also reported that its composition makes it soluble in a single solvent, glacial acetic acid [9]. The lower relative permittivity of glacial acetic acid enables it to dissolve kafirin by reducing inter-molecular interaction, unfolding the protein, and, consequently, improving solubilisation.

Various modifications such as physical treatment and chemical and enzymatic processing have been applied to improve the solubility of kafirin and zein. Any manipulation in the number and nature of cross-linking will influence the solubility of the protein [75].

Commonly, acid or alkali solutions are used to convert ionisable amino acid residues to acid or salt forms, a process called protein hydrolysis or protein denaturation. For example, in acid treatment kafirin is exposed to the acidic conditions, which protonates the basic amino acid residues., i.e., gaining of an electron to form positively charged ions. Similarly, acidic amino acid residues might also undergo protonation, forming negatively charged ions. Thus, the net influence of acid treatment is the conversation of ionisable amino acid residues into their respective acid or salt forms; thereby disrupting the native structure of kafirin, leading to denaturation and an increase in solubility.

In addition to chemical treatments, enzymatic modification is another prominent methodology to alter the solubility of prolamin proteins—or example, the solubility of zein increases when treated with glutaminase, which catalyses a deamidation reaction [76], and by protease and papain, which causes hydrolysis [77]. Deamidation is the hydrolysis of amide groups of asparagine or glutamine to aspartic and glutamic acid. Prolamins contain significant amounts of glutamine and asparagine that are susceptible to hydrolysis [75]. The increase in the solubility of prolamins occurs from the conversion of the amide group to the carboxylic acid group, which causes a protein to unfold and increases the surface charge of the protein. Yong et al. [76] reported the deamidation of α-zein in 70% ethanol by glutaminase. Spectroscopic analysis revealed less α-helix content, weakened aggregation of modified α-zein, and a subsequent increase in solubility. An increase in the solubility of zein protein was seen following enzymatic hydrolysis by alcalase and protease [77]. The authors reported native zein was insoluble in water; but, upon treatment with alcalase and protease the solubility increased to 69% and 45%, respectively. This increase in solubility is a result of polypeptide fragmentation that forms zein hydrolysate micelles.

Sonication treatment is an emerging technology that can be used to improve the solubility of kafirin. Via this cavitation process, unfolding and breaking of peptide bonds leads to exposing hydrophilic amino acid residues and, consequently, increasing its solubility [78]. Some plant proteins, including kafirin, have been subjected to dual modification, such as sonication in combination with alkali and acid treatment, which has led to an increase in solubility. [79]. Other technologies that may enhance the solubility of kafirin are high-pressure, pulsed electric field and gamma irradiation. Infrared and microwave irradiation has also been used to improve the solubility of kafirin protein [80]. They reported that irradiation lowered the hydrophobicity and increased the solubility of kafirin. These treatments caused changes in the primary and secondary structures of kafirin (e.g., a ratio of α helix to β sheet), and morphological analysis showed a distorted surface structure.

The insolubility of kafirin contributes greatly to its structural stability and mechanical properties, but may hinder morphological control during biomaterial production—for example, non-homogeneous dispersion during electrospinning leading to poor wetting of substrates [81]. Indeed, solubility of kafirin limits its application in aqueous alcohol or in glacial acetic acid, but it offers innovative ways to produce kafirin-based biomaterial. Factors such as solvent concentration, pH sensitivity, and denaturation behaviour should be carefully considered to optimise biomaterial production efficiency and to improve the performance of the encapsulate.

### 5.5. Surface Activity of Kafirin Protein

#### 5.5.1. Foam Formation

The foam forming capacity of the protein can be utilised to develop novel biomaterials with tailored porosity and mechanical properties. Proteins can have the capacity to adsorb at the interface, which promotes foam formation. Foam is the dispersion of air in a continuous liquid phase [82]. To act as a foaming agent, kafirin should rapidly adsorb at the air–water interface, undergo rapid conformational changes leading to re-arrangement, and form a cohesive viscoelastic film around bubbles [83]. Amphiphilic proteins can stabilise the foam interface via electrostatic forces and covalent linkage but have limited stability because of coalescence [83,84]. Therefore, foam forming capacity and protein stability are important quality parameters as they directly impact the structural integrity and functional properties of the biomaterial.

Because of a higher cysteine content, β- and γ-subunits of kafirin can form superior foams compared to α-kafirin [85]. These cysteine-rich subunits can form many elastic lamellae around air bubbles due to strong disulfide bonding [86]. High heat treatment and deamidation are prominent treatments that alter structural functionality and, subsequently, foaming capacity [87,88]. Teklehaimanot et al. [89] reported an abrupt breakdown of unmodified zein and kafirin foam immediately after agitation was stopped. However, they found that deamidation of the protein with 0.1 M NaOH resulted in stable foam formation. This increase in foaming capacity after deamidation results from the unfolding of the α-helix, leading to amphiphilic molecules (dominated by hydrophobic moieties) with surface activity. Riha et al. [90] extensively reviewed the deamidation of proteins. They stated that deamidation results in the reduction of amide side groups, while it promotes electrostatic repulsion and hydrophobic interactions, which, in turn, leads to unfolding of the α-helix structure.

#### 5.5.2. Emulsion Formation

Emulsions, characterised by the dispersion of one immiscible liquid phase within another, holds great promise for the development of biomaterials due to their versatility in encapsulating bioactive compounds to facilitate controlled release in an efficient manner [91]. Given the self-assembling properties of kafirin protein in water–alcohol mixtures, which leads to the formation of colloidal particles, Pickering emulsion have been used to improve stability [92]. The Pickering emulsion was developed in 1907 by Pickering [93] and is an oil-in-water or water-in-oil emulsion that is stabilised by solid particles that partially adsorb onto the interface between the continuous and dispersed phases to form steric tension (a mechanical barrier) that subsequently protects the emulsion droplets against coalescence [94]. The Pickering emulsions differ from the conventional emulsions primarily in terms of their stabilisation mechanism, i.e., reliance on solid particles as stabilising agents. The general properties for kafirin particles to act as Pickering emulsion stabilisers include (a) the partial wetting of kafirin particles in both phases whist remaining insoluble, (b) the wettability of kafirin particles should have ability to migrate to and reside at the interface, where they contribute to stabilisation of the emulsion, and (c) the size of kafirin particles should be smaller than the emulsion size (e.g., nano-range particles to stabilise sub-micron droplets) [92]. These emulsions have gained interest because of the use of natural stabilising agents and their excellent resilience to coalescence.

Because of its unique chemical nature, kafirin can be used as a stabiliser for Pickering emulsions. Kafirin’s properties—soluble in aqueous alcohols or acids but insoluble in water, high self-assembling capacity, easy to modify for particle formation, and low digestibility—make it suitable for this type of emulsion. Kafirin nano spherical particles were prepared as stabilising agents to develop Pickering emulsions (single water-in-oil) without any synthetic stabilising agents [92]. The authors reported kafirin-based oil-in-water emulsions exhibited resistance against coalescence. They proposed that the high resistance against the coalescence of kafirin-based Pickering emulsion is a combination effect of (a) the adsorption of the kafirin particle layer to form a physical barrier between emulsion droplets, consequently reducing coalescence, and (b) the lower zeta potential of kafirin particles in emulsion phases, suggesting electrostatic repulsive forces are stronger than attractive forces to prevent coalescence.

Single water-in-oil or oil-in-water emulsions are being replaced for enhanced stability by double emulsions of water-in-oil-in-water because of properties like higher masking of flavours and step-by-step compartments for delivery of both oil-soluble- and water-soluble-sensitive ingredients in a single formulation [95,96]. The formulated kafirin-based Pickering double emulsion (water-in-oil-in-water W_1_/O/W_2_) was developed and contains an inner aqueous phase (W_1_, made of gelatine containing anthocyanin in sodium citrate and citric acid buffer), an oil phase (O, made of soybean oil containing polyglycerol polyricinoleate), and an external water phase (W_2_ made of kafirin nanoparticles [97]. The water phase structure of the kafirin could be characterised by its hydrophobic nature, i.e., tendency to form insoluble aggregates in water. Thus, the structure of kafirin in the double emulsion is a localisation of kafirin molecules at the interface between internal droplets and the surrounding oil phase, leading to stabilisation of the system. The studies of kafirin-based emulsions reported success in development through careful tuning of the formulation parameters such as droplet size and release kinetics.

### 5.6. Dispersibility of Kafirin Protein

Dispersibility is the uniform distribution of protein particles in solution and depends on the nature of the protein and solvent system in use [98]. Kafirin and zein have poor dispersibility because of their isoelectric points of pH 6.2 [99] and pH 5.8 [100], respectively, at which the proteins precipitate and form aggregates [101,102]. Electrostatic interactions drive these aggregations; therefore, synthetic stabilising agents have been used to cause repulsive forces between the polymer chains, which prevents the particles from aggregation.

Food biopolymers have gained attention as stabilisation agents with prolamins because of clean label and sustainable profile. For example, sodium caseinate with zein [101] was used to improve the dispersibility of spray-dried zein particles, through its improved hydration and dispersibility, reduced turbidity, and increased stability/reduced precipitation at neutral pH to maintain structural integrity. The authors revealed that the addition of amphiphilic protein to zein reduced the surface hydrophobicity and increased the steric and electrostatic repulsion of zein, which minimised particle aggregation. A method for precipitation of zein using sodium caseinate to stabilise the zein colloidal suspension has been reported [103]. The formulated zein nanoparticles with sodium caseinate showed improved dispersibility and stability at pH 7.4 compared to zein nanoparticles without sodium caseinate.

A significant number of studies demonstrate improved protein colloidal stability using food gums [104,105,106]. These protein–gum interactions are driven by non-covalent electrostatic interactions in which the negatively charged carboxylate group of the gum contributes to the anionic charge, allowing electrostatic binding. [107,108]. For example, gum Arabic [109] has been shown to form stable complexes with zein proteins. Therefore, combining food gums with hydrophobic protein will increase steric repulsion within the protein molecule and, thereby, improve their pH, salt, and temperature stability. In these co-polymer dispersion systems, hydrophobic interactions, van der Waals interactions, dipole interactions, and Coulomb’s interactions between two counter-charging polymers result in the formation of polyelectrolyte complexes. These complexes result in optically homogeneous and stable nano-dispersion with dimensions in a colloidal size range. The main advantages of polyelectrolyte formation are the lower use of chemical cross-linking agents, reduced toxicity, biodegradability, less undesirable effects of the regents, and an acceptable taste. These polyelectrolyte complexes are categorised into two classes: stoichiometric and non-stoichiometric. In the stoichiometric class, polyelectrolyte complexes are prepared by the biopolymer, which is in an equimolar ratio; in non-stoichiometric polyelectrolyte complexes, one polymer is in excess to another. The mechanism of formation of polyelectrolyte complexes can be viewed as two counteracting processes [110]. First, electrostatic charge compensation leads to the ordering of two oppositely charged polyions into a complex molecule. Second, polyanions and polycations undergo chaotic aggregation. Importantly, during this chaotic aggregation, some ionic sites are still charge-compensated by the low-molecular-weight counterions. The factors that affect the formation and stability of polyelectrolyte complexes include charge-to-charge stoichiometry, charge density, molecular weight of biopolymers, polyelectrolyte concentration, pH, and ionic strength [110].

Using electrostatic interactions calcium alginate and zein particles were fabricated [111]. The formulated stable dispersion resulted in the controllable core–shell arrangement in which zein formed a shell and calcium alginate was entrapped within. The authors reported that the calcium alginate solution showed a more negative zeta potential, suggesting its high anionic character and the solution of zein protein resulted in a positive charge suggesting its cationic character. They reported that a complex of net negative charge was created with the addition of calcium alginate to the zein solution. This indicates that the overall negative charge (anionic groups) of the formulated system was significantly higher than the positive charge (cationic groups). Gali et al. [112] stabilised zein colloidal suspensions using gum Arabic for the encapsulation of rutin, a plant flavonol. A ratio of 1:1 zein to gum Arabic reduced the instability of the colloidal formulation by depositing a negatively charged layer, suggesting electrostatic interactions between negatively charged gum and positively charged zein. In addition, there was no precipitation of the colloid due to bridging flocculation between particles.

This comprehensive knowledge of the physicochemical properties of kafirin will not only enable researchers to design kafirin-based biomaterials with enhanced stability and functionality but will also facilitate their translation from the laboratory to the commercial world. The properties such as high hydrophobicity, water insolubility, low digestibility, surface morphology, and poor dispersibility of kafirin should be considered to meet the specific requirements.

## 6. Conclusions

Kafirin is a major protein component in sorghum grain and in sorghum bioethanol production waste. Kafirin is a unique and complex protein. It is usually extracted using aqueous alcohol mixtures or acetic acid methodology. Current knowledge of kafirin at the molecular level is that it contains three subtypes α-, β-, and γ kafirin. Among these subunits, α-kafirin is the most dominant, made of α-helices, β-sheets, and some random coils. Furthermore, kafirin assembles into the spherical particles following evaporation-induced self-assembly, which serves as a driving force to extensively study kafirin for potential development of biomaterials. Kafirin protein has shown excellent physicochemical properties that are required for biomaterials including an evaporation-induced self-assembling capacity, high hydrophobicity, low digestibility, and water insolubility. There are still some challenges associated with the development of kafirin-based biomaterials including (a) scalability issues of extraction and purification processes and (b) limited understanding of the structure–functionality relationship of kafirin need for optimal for biomaterial preparation. To overcome these challenges, researchers could utilise more advanced extraction technology and fully optimise the process using engineering approaches such as Design of Experiment using Response Surface Methodology. Additionally, advanced purification techniques such as chromatography could be utilised to enhance the purity and yield of kafirin and purifying individual subunits, which should have consistent and predictable functionality. An in-depth understanding of extraction methodologies and their impact on physicochemical properties of kafirin can provide newer strategies to develop kafirin-based biomaterials with tailored functionalities.

## Figures and Tables

**Table 3:** Amino acid profile of kafirin, composition, hydrophobicity of amino acid side chains, hydration capacity of amino acid residues, and water solubility of kafirin amino acids.

Amino Acid	^1^ Content in Total Kafirin (g/100 g)	^2^ Content in α-Kafirin (mol %)	^3^ Content in γ-Kafirin (mol %)	^4^ Hydrophobicity (kJ/mol) at 25 °C
Aspartic (Asp)	6.0 *–6.5 **	5.91	0	2.09
Threonine (Thr)	2.9 *–2.6 **	2.82	4.16	1.6
Serine (Ser)	4.3 *–41 **	4.98	4.89	−1.25
Glutamic acid (Glu)	28.2 *–30.0 **	23.80	14.18	2.09
Glycine (Gly)	1.4 *–1.1 **	0.67	4.95	0
Alanine (Ala)	11.8 *–12.4 **	11.87	6.22	2.09
Half-cysteine	3.2 *–0.4 **	0.36	7.05	NA
Valine (Val)	3.8 *–5.0 **	5.20	6.36	6.27
Methionine (Met)	2.1 *–1.0 **	1.77	1.74	5.43
Leucine (Leu)	17.5 *–19.2 **	17.48	11.20	9.61
Tyrosine (Tyr)	3.6 *–5.5 **	4.29	2.81	9.61
Phenylalanine (Phe)	6.6 *–6.4 **	4.40	1.92	10.45
Lysine (Lys)	0.1 *–0.1 **	0.89	0.57	NA
Proline (Pro)	10.2 *–10.0 **	7.50	19.65	10.85
Histidine (His)	1.6 *–0.9 **	2.30	9.03	NA
Arginine (Arg)	3.8 *–1.0 **	0.52	2.70	0
Isoleucine (Ile)	3.0 *–4.8 **	NA	NA	12.54

^1^ Data of amino acid composition reported from study of * Xiao et al. [41] and ** Jones et al. [47]; ^2^ Data of α-kafirin amino acid composition reported from Shull et al. [48]; ^3^ Data of γ-kafirin amino acid composition and Duodu et al. [23]; ^4^ Data of hydrophobicity reported from Fennema’s Food Chemistry [49].

**Table 5:** Difference in composition of amino acids between α-zein and α-kafirin at 22 kDa.

Amino Acids	*α*-Zein at 22 kDa	*α*-Kafirin at 22 kDa
Alanine	40	46
Arginine	3	2
Asparagine	14	16
Histidine	4	2
Leucine	48	46
Proline	22	21
Valine	15	17
Tyrosine	7	8
Tryptophan	0	1
Serine	19	14
Methionine	5	3
Glycine	3	2
Glutamine	50	56

Data of amino acid composition reported from Song et al. [55].

## Data Availability

No new data were created or analyzed in this study. Data sharing is not applicable to this article.
